# Organic–inorganic hybrid nanoflowers: types, characteristics, and future prospects

**DOI:** 10.1186/s12951-015-0118-0

**Published:** 2015-09-04

**Authors:** Seung Woo Lee, Seon Ah Cheon, Moon Il Kim, Tae Jung Park

**Affiliations:** Department of Chemistry, Chung-Ang University, 84 Heukseok-ro, Dongjak-gu, Seoul, 06974 Republic of Korea; Department of BioNano Technology, Gachon University, 1342 Seongnamdaero, Sujeong-gu, Seongnam-si, Gyeonggi-do 461-701 Republic of Korea

**Keywords:** Biosynthesis, Nanoflowers, Organic–inorganic hybrid

## Abstract

Organic–inorganic hybrid nanoflowers, a newly developed class of flower-like hybrid nanoparticles, have received much attention due to their simple synthesis, high efficiency, and enzyme stabilizing ability. This article covers, in detail, the types, structural features, mechanism of formation, and bio-related applications of hybrid nanoflowers. The five major types of hybrid nanoflowers are discussed, i.e., copper–protein, calcium–protein, and manganese–protein hybrid nanoflowers, copper–DNA hybrid nanoflowers, and capsular hybrid nanoflowers. The structural features of these nanoflowers, such as size, shape, and protein ratio generally determine their applications. Thus, the specific characteristics of hybrid nanoflowers are summarized in this review. The interfacial mechanism of nanoflower formation is examined in three steps: first, combination of metal ion and organic matter; second, formation of petals; third, growth of nanoflowers. The explanations provided herein can be utilized in the development of innovative approaches for the synthesis of hybrid nanoflowers for prospective development of a plethora of hybrid nanoflowers. The future prospects of hybrid nanoflowers in the biotechnology industry, medicine, sensing, and catalysis are also discussed.

## Background

Since the inceptive studies of new nanomaterials in the early 2000s, various nanomaterials with captivating morphologies such as the core–shell structure [1], and Janus particle [2] have been developed. Among these new nanostructures, the topographical features of nanoflowers have piqued the interests of scientists because the higher surface-to-volume ratio in comparison with spherical nanoparticles is beneficial to enhancement in the efficiency of surface reactions. Therefore, extensive studies focusing on the applications of the nanoflowers have been undertaken. Despite the increasing interest in nanoflowers, synthesis of these species requires very harsh conditions such as toxic organic solvents, high temperature, and high pressure. Thus, it is difficult to control their morphological features for tailoring the structures of nanoflowers, and it is a formidable task to apply these particles in the field of biochemistry.

Enzymes are biological species with excellent activity and substrate specificity, but their use is limited by certain drawbacks such as high sensitivity to the surrounding environment, low reproducibility of experimental outcomes, and the requirement for complex purification processes. To facilitate application in drug delivery systems or detectors, novel nanomaterials called “nano-biomaterials” have been developed. These are also known as “organic–inorganic hybrid nanomaterials;” the name indicates that all of the inorganic nanoparticle components are bound to organic materials. Current methods for the preparation of nano-biomaterials can be classified into four categories in terms of immobilization [3–6], conjugation [7–9], crosslinking [10–13], and self-assembly [14]. Nano-biomaterials have numerous prospective applications in catalysis [15–18], biosensors [19–22], and drug delivery [23–26].

Proteins as enzymes have a strong affinity for metal ions as cofactor and thus, the stability of proteins is generally enhanced during immobilization onto the metal surface by the interaction such as charge affinity, covalent bond, and structural coupling [27, 28]. However, immobilized proteins exhibit lower activity than the free enzyme, primarily due to the loss of their activity by changing the orientation during the immobilization process and mass-transfer limitations on solid supports [29–32]. Single-protein nanoparticles and nanogels are examples of novel nano-biomaterials that have been used as catalysts with highly preserved activities [3, 10–13]. The activity of enzymes in these nanoparticles and nanogels could be as much as ~60–90 % of that of the free enzymes. In one of the few examples where the activity is better than that of the free enzyme, organophosphorus hydrolase embedded in mesoporous silica demonstrated an activity of ~200 % in solution compared with the free hydrolase [33]. In the case of trypsin, which is a hydrolyzing enzyme that can digest itself, its immobilization on a solid support increased the catalytic efficiency by thousands of times compared with that of free trypsin [34]. However, problems such as loss of enzyme properties are still encountered in these complicated processes.

Recently, a new approach for facile and safe synthesis of hybrid nanomaterials was developed to overcome the limitations of conventional methods [35]. Because it is possible to fabricate a nanomaterial simply by adding protein to metal ion solution, this synthetic method does not require any toxic elements or extreme harsh conditions. Therefore, the organic substance involved in the synthesis is subjected to less manipulation compared with other conventional methods to maintain the activity of the immobilized enzyme.

The flower-like hybrid nanomaterials generated by this process are called “organic–inorganic hybrid nanoflowers” or “hybrid nanoflowers”. Their synthesis mechanism, physical properties, protein activity, stability, and reproducibility have been intensively studied, and thus far, these species have exhibited significantly better properties than the free enzymes. The feasibility of in vivo application of nanoflowers in protein complexes, medicines, and serological studies is also a topic of investigation. However, a new paradigm shift is required in application of hybrid nanoflowers from enzyme stability improvements and efficient drug delivery systems to new horizons such as cell imaging, biosensor, and medical approaches.

This review presents an overview of several synthetic strategies that have been suggested to diversify nanoflowers, including their structural features. Moreover, the synthesis mechanism is confirmed through analysis. On the basis of experimental and theoretical data, we propose future prospects for novel nanoflowers and their further enhancement.

### Types of nanoflowers

Hybrid nanoflowers can be classified on the basis of the structure of the particles and the type of metal and organic material used. The classifications are summarized in Table 1.

**Table 1:** Types of organic–inorganic hybrid nanoflowers

	Metal ion	Organic material	Target	References
1	Copper	α-Lactalbumin	–	[35]
		Laccase	Catecholamines, phenols	
		Carbonic anhydrase		
		Lipase		
2	Copper	Laccase	Phenol	[36]
3	Copper	Glucose oxidase and HRP^a^	Glucose	[37]
4	Copper	Trypsin	HRP^a^, BSA^b^	[38]
5	Copper	HRP^a^	Hydrogen peroxide, phenol	[39]
6	Calcium	α-Amylase	Cnp-G3^c^	[44]
7	Calcium	Chitosan	Hydrogen peroxide	[45]
8	Manganese	Immunoglobulin G	Ractopamine	[46]
		Anti-ractopamine ab		
		Bovine serum albumin		
9	Copper	DNA	Specific cell	[55]
10	Copper	Bovine liver catalase	Hydrogen peroxide	[56]

^a^Horseradish peroxidase

^b^Bovine serum albumin

^c^2-Chloro-4-nitrophenylmaltotrioside

#### General nanoflowers using copper(II) ions and proteins

Since the initial development of hybrid nanoflowers consisting of copper(II) ions and proteins, these species have been intensively studied by focusing on their efficiency and stability [35–39]. Because many studies on copper–protein hybrid nanoflowers have been investigated well than those of other hybrid nanoflowers, the synthesis mechanism and applications of copper–protein hybrid nanoflowers are relatively well understood.

Ge et al. [35] first serendipitously discovered the hybrid nanoflower and confirmed that the copper ion and protein create a new type of particle via interaction. Subsequently, they synthesized four types of hybrid nanoflowers using α-lactalbumin, laccase, carbonic anhydrase, and lipase, respectively. The synthesized hybrid nanoflowers were used in the detection of phenols and oxidation of catecholamines. Each efficiency of the nanoflowers was found to be the same or superior to (95–650 %) to those of the conventional free enzyme solutions. The increased efficiency is derived from the following interplay of factors: (i) the large surface area of the nanoflower which does not cause mass-transfer limitations; (ii) the cooperative interaction of the entrapped enzyme molecules; (iii) the mutual influence of the enzyme and the microenvironment of the nanoflower that contains metal ions on each other (for example, Cu^2+^ ions in the nanoflowers may enhance the activity of laccase).

**Fig. 1:**
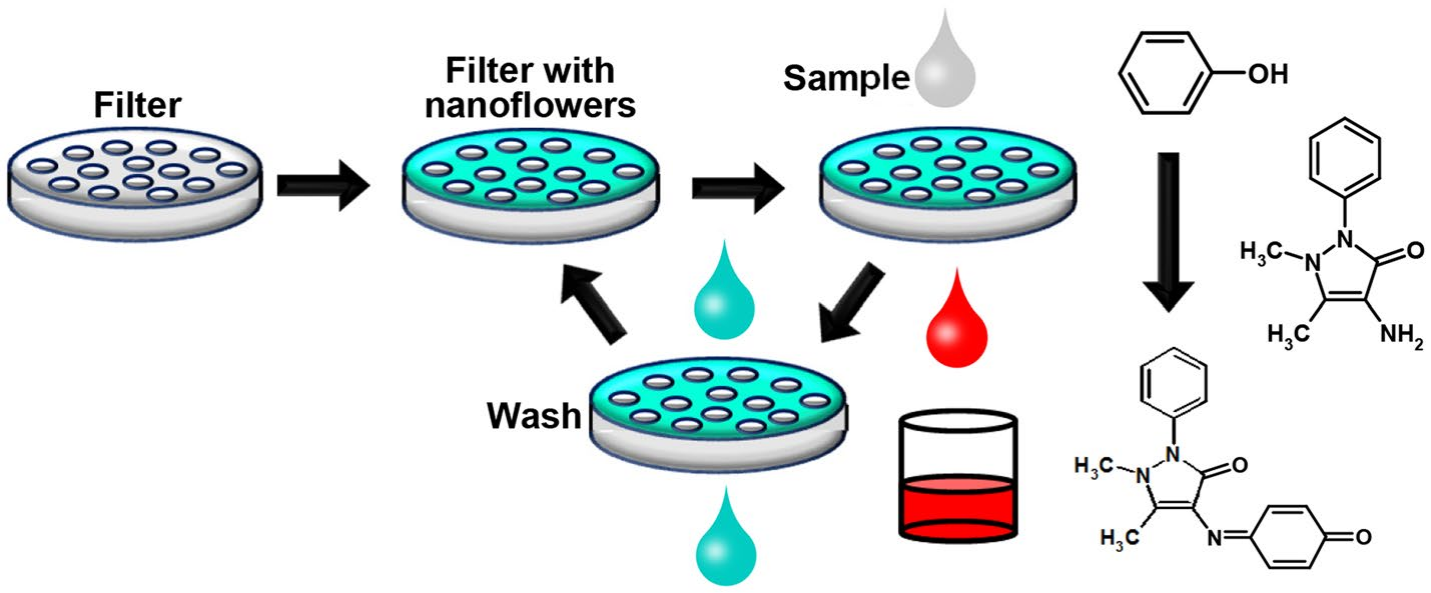
Application cycle of the membrane with incorporated laccase nanoflowers: fabrication, use, rinse, and reuse. Phenol and *ortho*-, *meta*-, and *para*-substituted phenols react with 4-aminoantipyrine to form colored compounds, which can be easily detected. Reproduced with permission from Ref. [36]: Copyright 2013 Wiley-VCH Verlag GmbH & Co. KGaA, Weinheim

Furthermore, the researchers developed a simplified phenol detector comprising a syringe filter containing adsorbed hybrid nanoflowers of laccase [36] (Fig. 1). Briefly, a mixture of aqueous phenol and 4-aminoantipyrine was injected into the nanoflower-coated filter using a syringe and kept in the filter chamber for 5 min. The high activity of laccase entrapped in the nanoflowers enabled rapid oxidative interaction of phenol with 4-aminoantipyrine to generate antipyrine dyes. The final red solution was pushed out of the syringe and collected for analysis by naked-eye visualization or by using a UV/Vis spectrophotometer. The detection rate of this method is faster than that of pre-existing gas chromatography (GC) or liquid chromatography (LC). This method also facilitated the reuse of the filter for about 1 month through cleaning due to the high stability of the enzyme in the particles.

**Fig. 2:**
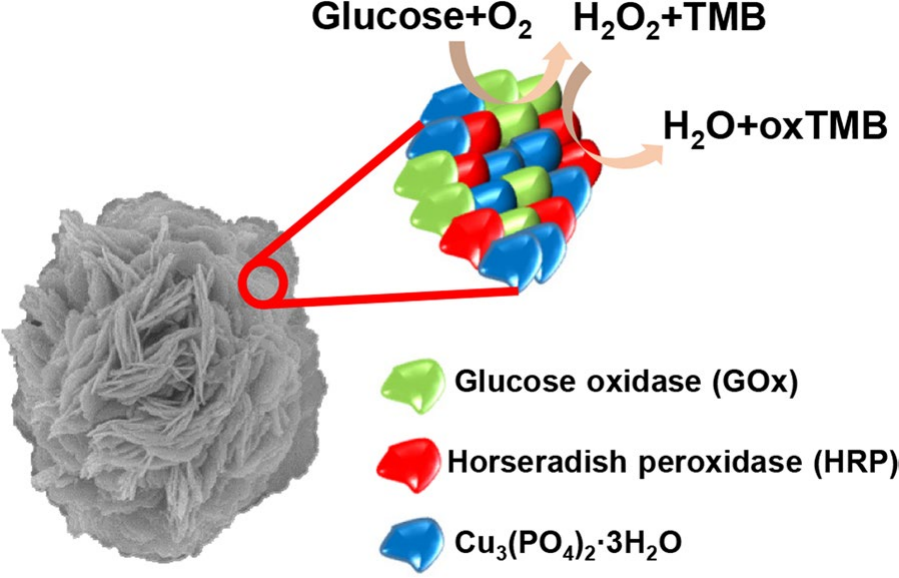
The cascade enzymatic reaction of multi-enzyme co-embedded hybrid nanoflower for glucose detection. Reproduced with permission from Ref. [37]: Copyright 2014, Royal Society of Chemistry

Following this germinal research, Sun et al. synthesized multienzymatic hybrid nanoflowers using glucose oxidase (GOx) and horse-radish peroxidase (HRP) [37], demonstrating the possibility that two or more enzymes could be included in a single hybrid nanoflower. Even to date, most co-immobilizations of multi-enzyme are performed via comparatively complicated processes such as by covalent cross-linking [40], encapsulation [41], gene fusion [42], and post-translational enzyme conjugation [43]. Unfortunately, these techniques are time consuming or result in reduced enzymatic activity, which limits the application of the hybrid materials. As an alternative, the discovery of this novel nanoflower offers a fast and simple synthesis. The procedure used in this experiment (Fig. 2) is as follows: glucose oxidized by GOx generates H_2_O_2_, which is immediately catalyzed by HRP to oxidize 3,3′,5,5′-tetramethylbenzidine (TMB), resulting in a color change from clear to blue. The conventional two-step process generally does not lead to high efficiency owing to the diffusion of H_2_O_2_ generated from glucose oxidation. However, in the hybrid nanoflower, loss of the generated H_2_O_2_ can be reduced because GOx and HRP are simultaneously present, resulting in more accurate detection.

The trypsin hybrid nanoflower synthesized by Lin et al. was used for enhanced protein analysis [38]. Mass spectrometry (MS) used widely for proteomic analysis, involves analysis of enzymatically digested peptides created from the parent protein. Therefore, effective proteolysis is required as a key step for effective identification of proteins. Generally, proteolysis is performed by using free enzymes, and suffers from some limitations such as long digestion time, autolysis, low stability towards environmental changes, and difficult recovery of products. Enzymes immobilized on solid supports have been used to overcome these disadvantages. The trypsin hybrid nanoflower was first used as an immobilized enzyme by Lin et al. to achieve high proteolytic performance, good enzyme stability and reusability, and a short digestion time [38]. Moreover, the activity of the enzyme component of the hybrid nanoflower was comparable to that achieved in various conventional protein analyses. The favorable results indicate that the hybrid nanoflower is a promising enzyme system for proteomic analysis.

A hybrid nanoflower employing copper ions and HRP for visual detection of hydrogen peroxide and phenol was also synthesized [39]. As above, the hybrid nanoflower was used as a catalyst in the reaction of hydrogen peroxide and phenol in the presence of 4-aminoantipyrine. Changes in the solution color could be detected with concentrations as low as 0.5 μM hydrogen peroxide and 1 μM phenol by the naked eye. The threshold concentration of hydrogen peroxide to prevent cellular damage is 50 μM; thus, the limit of detection (LOD) obtained with the use of the hybrid nanoflower meets the requirement for early clinical diagnosis. The LOD for phenol also satisfies the requirements for application to the detection of water pollution. In contrast, the LODs for hydrogen peroxide and phenol using free HRP were ~20 and ~10 μM, respectively. The enzyme activity of the HRP hybrid nanoflower is enhanced by the large surface-to-volume ratio that facilitates contact with reactants. Moreover, the reaction time of the HRP hybrid nanoflower (~5 min) is faster than that of free HRP (~25 min). The above results revealed that the hybrid nanoflower is more useful for the development of a highly sensitive colorimetric sensing platform.

#### General nanoflowers using calcium(II) ions and proteins

**Fig. 3:**
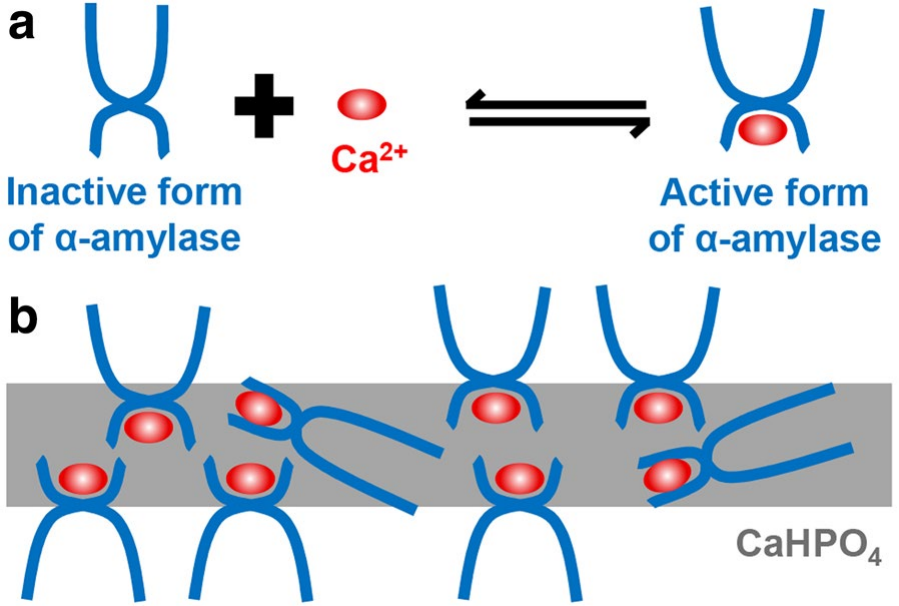
**a** Allosteric effect of α-amylase: α-amylase changes from inactive form to active form by binding calcium ion to allosteric site in inactive α-amylase and **b** α-amylase immobilized in nanocrystals. Reproduced with permission from Ref. [44]: Copyright 2013, American Chemical Society

Nanoflowers employing calcium ions have also been investigated, even though most nanoflowers are synthesized with the copper ion [44, 45]. A nanoflower employing calcium phosphate crystals was synthesized by Wang et al. by using the same method used for the synthesis of copper nanoflowers [44]. The flower-like morphology was confirmed and a postulate of how the activity increases due to the enzyme immobilization procedure was presented. The α-amylase used in this experiment is an enzyme that exhibits allosteric phenomena (Fig. 3a). In the absence of calcium ions, α-amylases are usually in an inactive state as the functional site is inhibited. In contrast, in the presence of calcium ions, the allosteric sites of α-amylase are occupied by the calcium ion, thus affecting the structure of the functional site. During the synthesis of the hybrid nanoflower using calcium ions and α-amylase, the calcium ions activate and are strongly bound to α-amylase. Because the two elements are located close to each other during the formation of the hybrid nanoflower, α-amylase was active for a longer period than the free enzyme, which is randomly and briefly activated by calcium ions (Fig. 3b). Thus, Wang et al. showed that it is essential to understand the chemical interaction between the protein and nanomaterials to improve the functionality of the protein in a given environment [44].

**Fig. 4:**
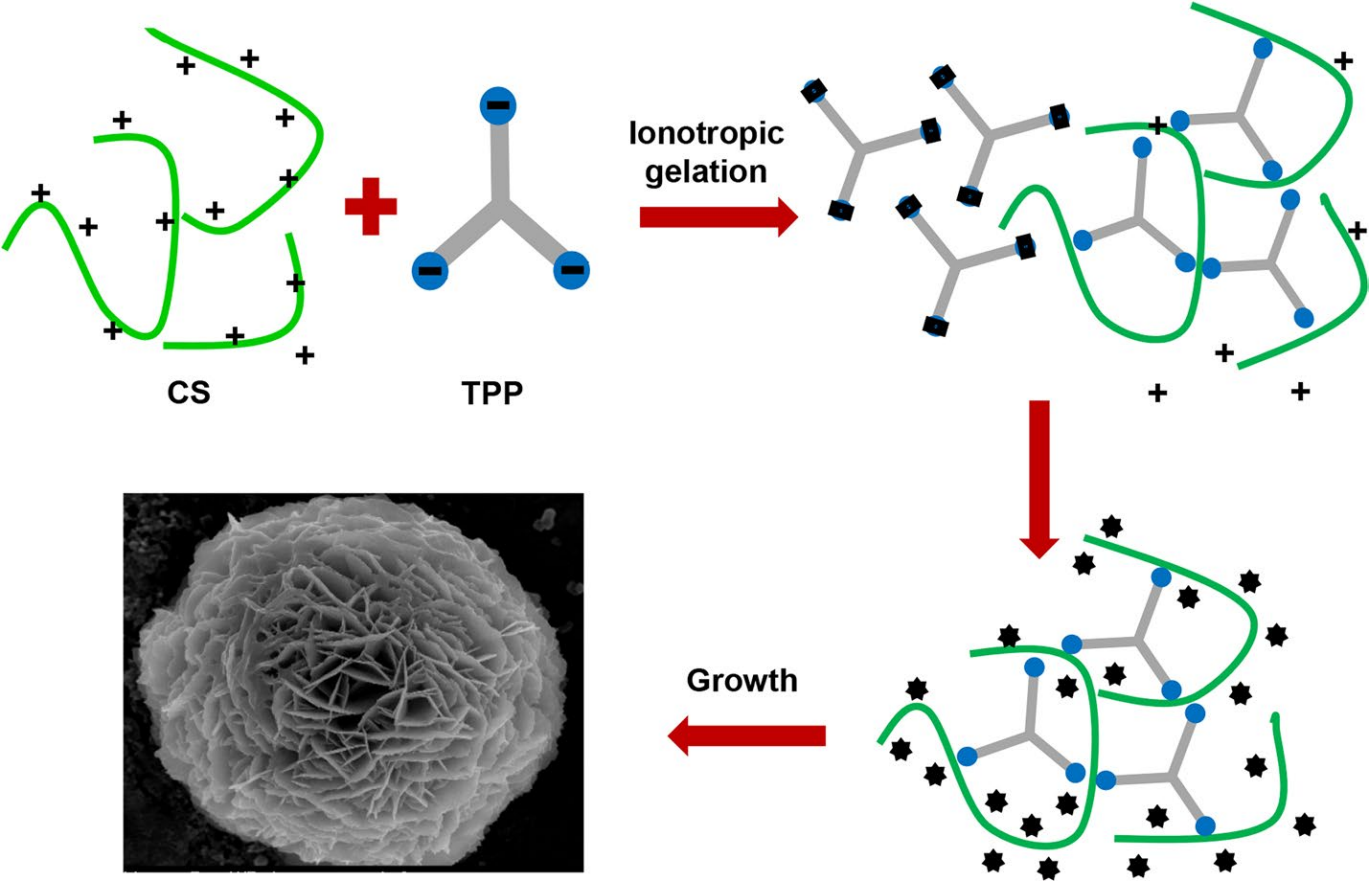
Schematic synthesis of chitosan–calcium ion hybrid nanoflower. Chitosan binds to pyrophosphate through ionotropic gelation and generates CS-TPP gel complex which reacts with calcium phosphate crystal to form hybrid nanoflower. Reproduced with permission from Ref. [45]: Copyright 2014, American Chemical Society

A hybrid nanoflower employing calcium ions was also presented in a recent study [45]. Unlike previous synthesis methods using protein solution, chitosan (CS) and tripolyphosphate (TPP) were used to fabricate a gel-form composite via ionic bonding. Although this method has different steps from that used by Hou et al. [44], the mechanism of nanoflower formation is the same (Fig. 4). Calcium ions and TPP form calcium phosphate crystals, and the nanoflower is obtained by the reaction of these crystals and the CS-TPP gel complex. Almost any type of catalyst could be used in this experiment because the catalyst combines with chitosan in the CS-TPP gel complex by electrostatic interaction. However, because addition of the catalyst declined in the nucleation sites for calcium phosphate formation, it should be noted that the maximum amount of catalyst for the formation of the nanoflower was 5.0 mg. This technique for nanoflower synthesis provides a new approach that facilitates the generation of the nanoflower using a variety of organic substances.

#### General nanoflowers using manganese(II) ions and proteins

**Fig. 5:**
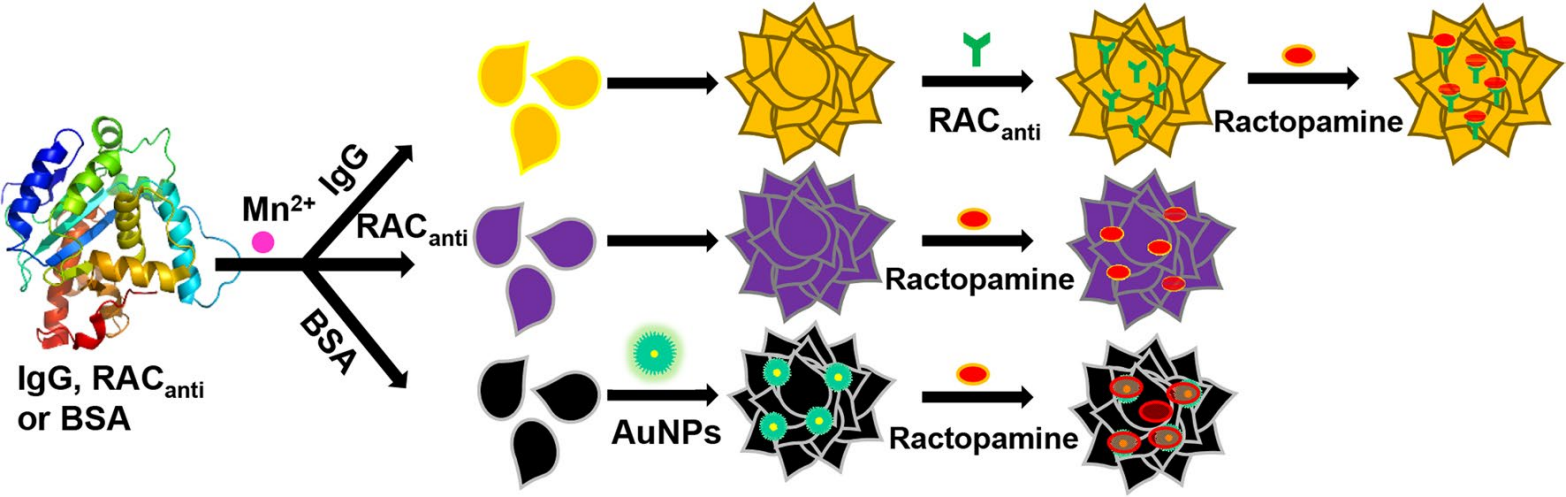
Schematic representation of the synthesis of manganese-based hybrid nanoflowers as a novel electrochemical biosensor for the detection of ractopamine, including (i) Mn_3_(PO_4_)_2_-IgG, (ii) Mn_3_(PO_4_)_2_-RAC_anti_ (anti-ractopamine antibody), and (iii) Mn_3_(PO_4_)_2_-BSA-Au nanoflowers. Reproduced with permission from Ref. [46]: Copyright 2015, Elsevier

Manganese phosphate hybrid nanoflowers were synthesized by Zhang et al. to apply into a novel electrochemical biosensor for detection of ractopamine, which can cause acute poisoning consumed by humans [46]. Although many analytical methods have been developed for the detection of ractopamine, such as liquid chromatography–mass spectrometry (LC–MS) [47, 48], gas chromatography–mass spectrometry (GC–MS) [49], high-performance liquid chromatography (HPLC) [50], and bioassay [51]. They have some limits owing to high instrument cost and long-time analysis. To solve the problem, electrochemical methods were applied by using the phenolic hydroxyl group, which makes ractopamine electrochemically active [52]. However, this detection method was also limited that due to its low response activity on electrode surfaces. Hybrid nanoflower as a novel electrochemical biosensor overcomes the disadvantage. These nanoflowers are made by manganese(II) ions and three types of protein, immunoglobulin G (IgG), ractopamine antibody (RACanti), and bovine serum albumin (BSA) (Fig. 5). The detection limits of the developed electrochemical biosensors were 4.6, 9.32, and 26 pg mL^−1^, respectively. Compared with the detection limits of previous different methods, these results present that electrochemical methods using hybrid nanoflowers are more sensitive [52–54]. This study shows that the hybrid nanoflower can be used as electrochemical tools as well as catalyst or drug delivery matter.

#### General nanoflowers using copper(II) ion and DNA

**Fig. 6:**
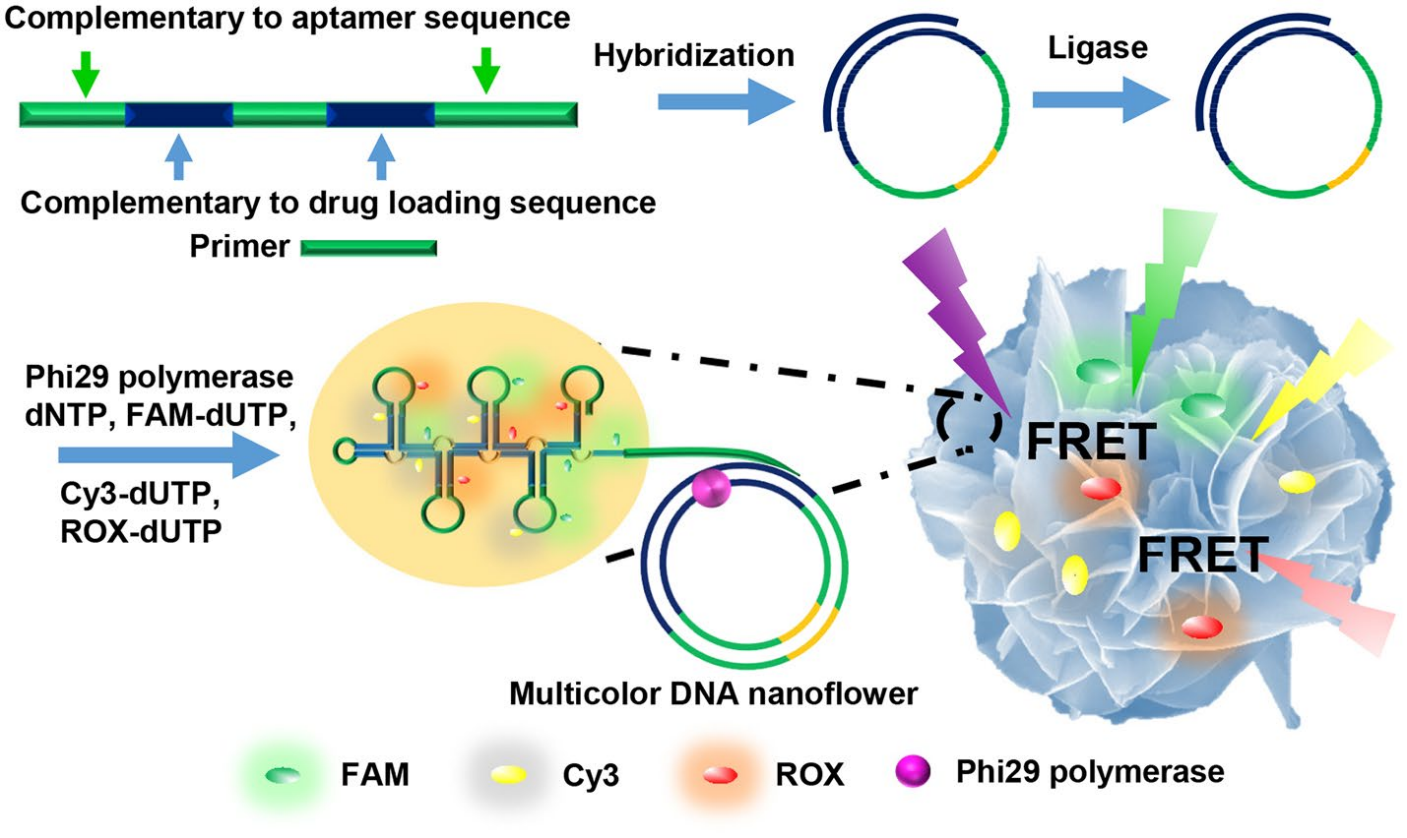
Sequence-independent self-assembly of multicolor FRET (fluorescence resonance energy transfer) DNA hybrid nanoflower. Reproduced with permission from Ref. [55]: Copyright 2014, Wiley-VCH Verlag

Hu et al. used DNA as the organic material instead of protein in hybrid nanoflowers [55]. Because DNA is highly soluble in aqueous medium and has a high content of nitrogen atoms in its structure like protein, it could be used in the synthesis of hybrid nanoflowers by binding metal ions. The DNA hybrid nanoflower morphology was combined with the fluorescence resonance energy transfer (FRET) phenomenon to obtain high-resolution images of cells or to use for traceable drug delivery systems. In brief, the researchers created a site in the DNA template for the simultaneous attachment of a drug and fluorescent dyes (FAM, CY3, ROX), and subsequently, they synthesized a hybrid nanoflower coupled to the fabricated DNA by rolling circle replication (RCR) with the metal ions (Fig. 6). Consequently, they were able to obtain a high-resolution image based on FRET between the dyes using long-wavelength light, which does not affect the cells. Furthermore, the path of drug delivery in the living cells was successfully traced by monitoring the light emitted by the dyes. Thus, Hu et al. suggested that DNA hybrid nanoflowers can be applied in many fields by showing the feasibility of the synthesis of DNA hybrid nanoflowers [55].

#### Capsular nanoflowers using copper(II) ions and proteins

**Fig. 7:**
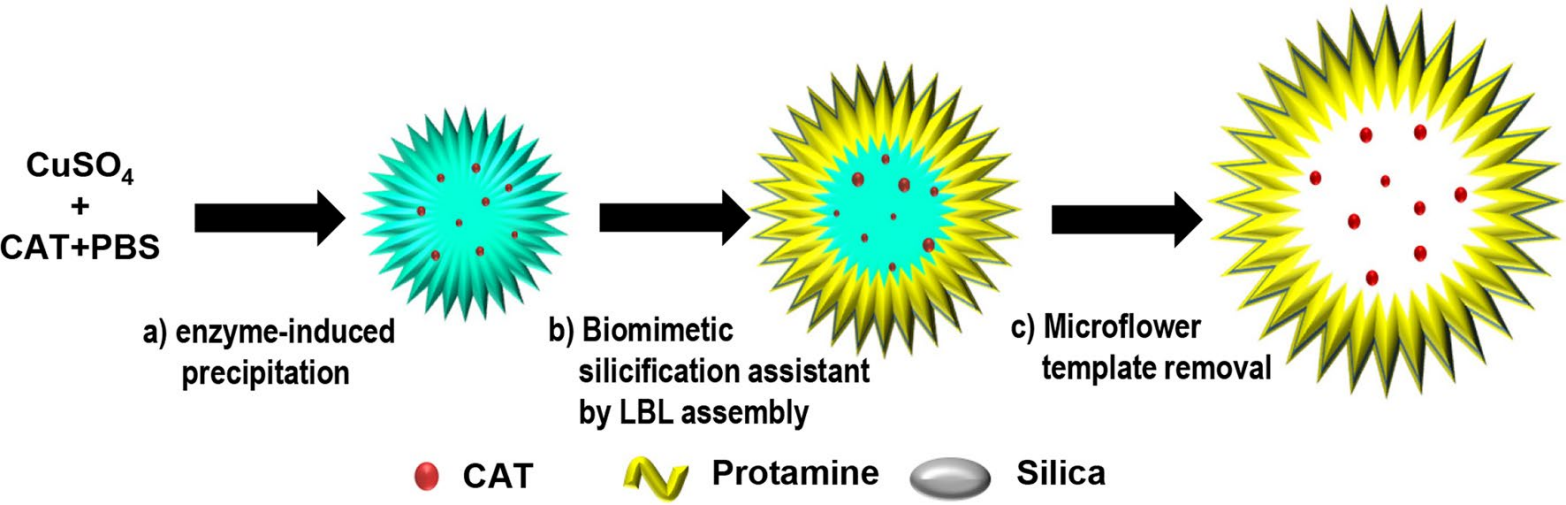
Scheme of preparation procedure of the FPSH capsules: **a** formation of protein–inorganic hybrid microflowers; **b** formation of (protamine-silica)_2_ bilayers on the microflowers; **c** formation of the FPSH capsules after eliminating the microflower template through EDTA treatment. Reproduced with permission from Ref. [56]: Copyright 2014, The Royal Society of Chemistry

A capsular nanoflower was synthesized by Jiang and co-workers, as shown in Fig. 7 [56, 57]. Compared with early hybrid nanoflowers synthesized from a metal phosphate and protein, the capsular nanoflower was synthesized by an additional wrapping with protamine and silica through a biomimetic mineralization approach and removing the metal from the core. Because the rough surface of the multiple shells facilitates the adsorption of substrates, the capsular nanoflower exhibited significantly enhanced enzyme activity as well as better stability under extreme conditions (high temperature, pH, long-term storage) than simple hybrid nanoflowers. These properties provide a practicable and useful solution for enhancing the efficiency in bio-chemical applications such as catalysis and drug delivery.

### Characteristics

#### Structural features and enzyme efficiency

As shown in Table 2, the hybrid nanoflowers can be classified based on characteristics such as shape, size, protein/total weight ratio percent, and enzyme efficiency compared to that of the free enzyme.

**Table 2:**
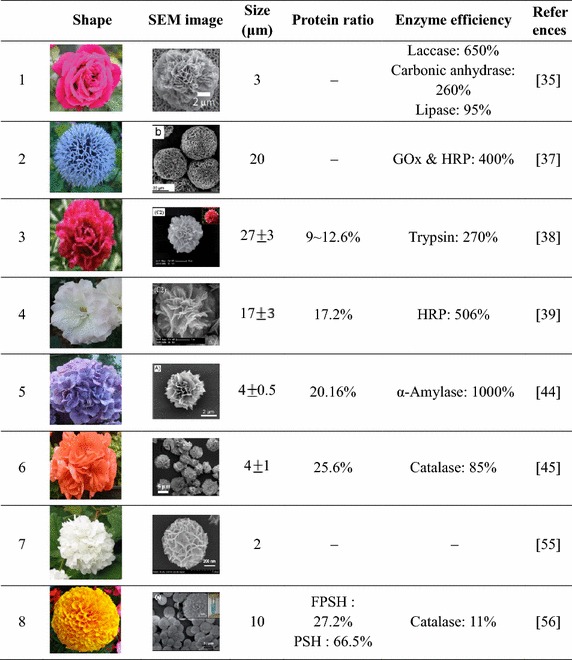
Characteristics of organic–inorganic hybrid nanoflowers

^a^Rose

^b^Blue ball

^c^Peony

^d^Satin

^e^Water flower

^f^Zonal

^g^Viburnum opulus

^h^Marigolds

Although the size of hybrid nanoflowers varies depending on the protein concentration, the range of variation is narrow (about ±3 μm). Thus, based on the average size from optimized data, the nanoflowers range from 2 to 30 μm, and the pore size (which is the diameter of the hole between petals) is approximately 0.1 μm. Because the size of most of these hybrid flowers is expressed in micro units (μm), we believe that the use of the term “microflower” is more correct than “nanoflower.” In addition, the shape of the hybrid nanoflowers is similar to that of round flowers, but with subtle variations. Thus, the pictures that resembles the respective scanning electron microscopy (SEM) images are provided to highlight the differences (Table 2).

The weight ratio of protein to total particle weight measured by thermogravimetric analysis (TGA) is also summarized in Table 2, demonstrating that the protein constitutes 10–66 % of the total weight. Furthermore, as more protein is used, the percentage of protein increases. However, the encapsulation efficiency (the ratio of the amount of immobilized protein to the total amount of protein employed) follows a completely opposite trend. Excessive addition of protein to a constant weight of inorganic component induces a dramatic decrease of the encapsulation efficiency. In light of these considerations, the proper amount of protein must be selected to conserve the protein and to attain good morphology and a high weight percentage.

Finally, we consider the enzyme efficiency of hybrid nanoflowers. Compared with the corresponding free enzyme solutions, the enzyme efficiency of hybrid nanoflowers varies from 85 to over 1000 %. These results suggest that the proteins in hybrid nanoflowers have higher activity and stability than the corresponding free enzyme solutions despite immobilization in the flower petals. Moreover, hybrid nanoflowers overcome the previously encountered problem of mass-transfer limitation and open up an avenue for application to various research and detection fields.

#### Mechanism

**Fig. 8:**
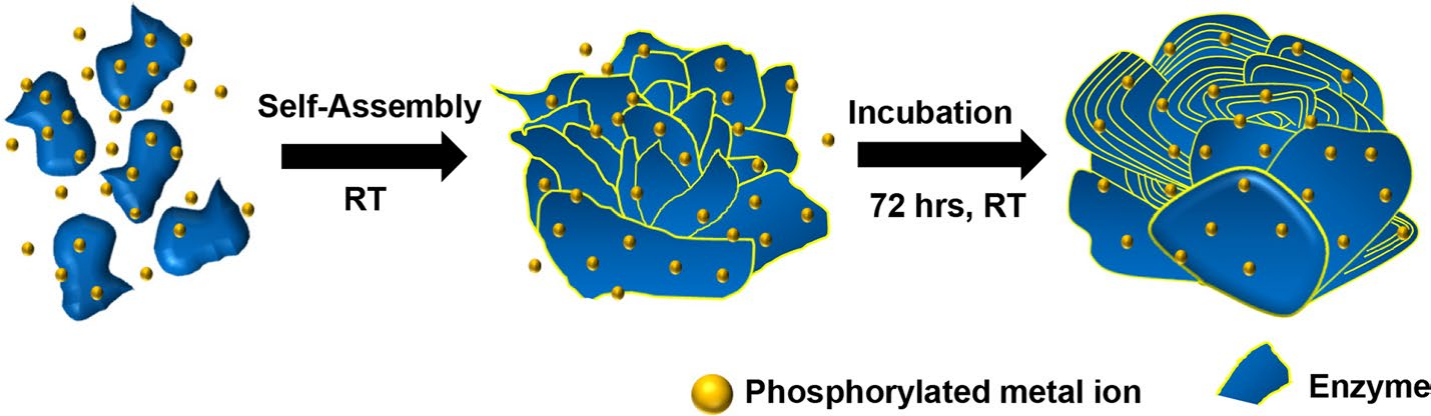
Synthesis mechanism of organic–inorganic hybrid nanoflower. Reproduced with permission from Ref. [35]: Copyright 2012, Nature Publishing Group

The mechanism for the synthesis of organic–inorganic hybrid nanoflowers comprises three steps (Fig. 8). In the early growth step, primary crystals of metal phosphate [M_3_(PO_4_)_2_, (M: Cu, Ca)] are formed. At this stage, the organic molecules (protein, DNA) form complexes with the metal ions (Cu^2+^, Ca^2+^), mainly through coordination via the amide groups in the protein backbone. These complexes provide a location for nucleation of the primary crystals. In the second growth stage, metal-protein crystals aggregate into large agglomerates of protein molecules and primary petals are formed. The kinetically controlled growth of metal phosphate crystals originates on the surfaces that have the Cu^2+^ binding sites of these agglomerates, causing flower-like petals to appear in the embryo. In the final step, anisotropic growth leads to complete formation of a branched flower-like structure. In this growth process, the protein induces nucleation of the metal phosphate crystals to form the scaffold for the petals and serves as a “glue” to bind the petals together. In the absence of the proteins, large crystals, but no nanoflowers, are formed.

### Future prospects

In the 1960s, the development of enzyme immobilization technology inspired chemists to design new biomaterials containing highly stabilized enzymes. However, immobilization generally led to loss of the enzyme activity. By the 1990s, the activity of enzymes immobilized on nano-sized materials could be maintained at levels up to that of the free enzymes. Interest in this field was again awakened with the unearthing of the first organic–inorganic hybrid nanoflower in 2012, and many studies focusing on new flower-like hybrid nanomaterials are still in progress.

Future research for the development of drug delivery systems, biosensors, biocatalysts, and bio-related devices is anticipated to take multiple directions. New synthesis principles, new types of hybrid nanoflowers, and detailed mechanisms are expected to emerge. The application of nanoflowers in bio-catalysis and enzyme mimetics, tissue engineering, and the design of highly sensitive bio-sensing kits, as well as industrial bio-related devices with advanced functions, various and controllable syntheses, biocompatibility, and modifications of hybrid nanoflower structures and properties, should receive increasing attention.

## Conclusions

In summary, organic–inorganic hybrid nanoflowers have piqued the interest of researches and numerous related papers have been published. Research in this field is spurred by the simplicity of the synthesis and safe conditions. Moreover, high efficiency and enzyme stability are readily achieved with hybrid nanoflowers. We believe that the study of organic–inorganic hybrid nanoflowers will lead to creative solutions and rapid development of biomaterials and biotechnology industries.

## Abbreviations

GOx: glucose oxidase
HRP: horse-radish peroxidase
LOD: limit of detection
CS: chitosan
TPP: tripolyphosphate
SEM: scanning electron microscopy
